# Foundational endovascular concepts and devices for cardiac surgeons

**DOI:** 10.1016/j.xjse.2025.100061

**Published:** 2025-07-07

**Authors:** Irbaz Hameed, Akbar Bazarbaev, Nyle Bajwa, Samantha Colon, Adham Ahmed, Harris Ahmad, Ayesha Mubasher, Sriharsha Talapaneni, Christopher K. Mehta, Marvin D. Atkins, Bradley Taylor

**Affiliations:** a Division of Cardiac Surgery, Department of Surgery, Yale University School of Medicine, New Haven, Conn; b Department of Cardiothoracic Surgery, Northwestern Medicine, Chicago, Ill; c Department of Cardiothoracic Surgery, Houston Methodist Hospital, Houston, Tex; d Department of Cardiothoracic Surgery, University of Maryland Medical System, Baltimore, Md

**Keywords:** endovascular, transcatheter, minimally-invasive, education

**Figure:**
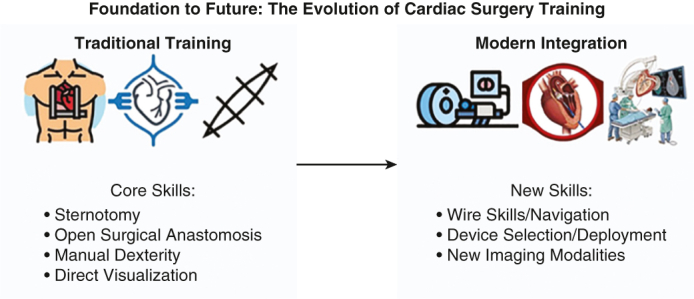
Foundation to future: the evolution of cardiac surgery training.

**Central Message**

As endovascular and minimally invasive interventions grow, it is critical for trainees and early career surgeons to develop expertise in these technologies to remain relevant in multidisciplinary care.

**Perspective**

This review introduces core endovascular surgery concepts and clinical applications relevant to contemporary multidisciplinary practice. We also review emerging endovascular devices to equip early surgeons with practical knowledge to collaborate with interventionalists in the hybrid operating room.

Cardiac surgery has traditionally been defined by open operations.^1^ The rapid advancement of endovascular technology, however, is transforming the field. From transcatheter valvular interventions to thoracic endovascular aortic repair (TEVAR), the modern cardiac surgeon's skillset now necessitates wire and catheter navigation, endovascular imaging, and percutaneous device handling. A fundamental understanding of these concepts is critical.^2^

This guide introduces foundational concepts in endovascular surgery in the context of the cardiac surgeon. The guide will equip readers with an understanding of guidewires, sheaths, catheters, balloons, and valves currently available, as well as the decision-making process that pairs specific devices with clinical scenarios. By assimilating these insights, trainees and early-career surgeons can integrate into multidisciplinary teams and meaningfully contribute to discussions.

## Foundational Concepts in Endovascular Therapy

### Catheter-Based Versus Surgical Approaches

Endovascular interventions rely on percutaneous vascular access, imaging guidance, and intraluminal tools. Instead of surgical incisions, wires and catheters are navigated through peripheral arteries or veins under fluoroscopy. The shift requires mastering a new visual-spatial approach, redirecting attention from the surgical field to radiology monitors and echocardiographic screens.^3^ Operators must also become comfortable with complex instrumentation, including large-bore sheaths, catheters, guidewires, and various devices.

### The Hybrid Operating Room

The hybrid operating room combines a sterile environment with advanced imaging capabilities. Fixed C-arms, biplane angiography systems, and integrated ultrasound and 3-dimensional (3D) imaging tools allow operators in this setting to concomitantly perform open surgery and complex endovascular interventions.^4^ Surgeons must learn to operate effectively in this environment, appreciating table movement, patient positioning, and imaging angle optimization.

### Radiation Safety

Intraprocedural radiation requires operators to balance optimal image quality while minimizing radiation exposure. Patient factors like body mass index can significantly impact exposure levels, with each unit increase leading to approximately 5% greater radiation dose. Protective measures include maximizing distance from the radiation source, reducing fluoroscopy frame rates, and limiting cine-loop acquisition as necessary. In addition, the use of lead aprons, thyroid shields, leaded glasses, and/or ceiling-mounted and table-side radiation shields can reduce scatter exposure by 80% to 90%.^5^ Modern systems are also equipped with advanced settings to reduce cumulative radiation exposure such as pulsed fluoroscopy.^6^

### Multidisciplinary Heart Team

Collaborative decision-making with other specialties is key to optimizing patient selection and outcomes. Accordingly, several societal guidelines advocate for a Multidisciplinary Heart Team,^7^ including surgeons, interventionalists, cardiologists, electrophysiologists, cardiac anesthesiologists, and advanced care practitioners when evaluating patients for interventions.

## Vascular Access and Closure

### Common Access Sites

Common femoral artery (CFA) access is preferred for large-bore devices. During preoperative planning, computed tomography (CT) helps assess iliofemoral size, calcifications, and tortuosity to guide delivery sheath selection and reduce vascular complications.^8^ The use of ultrasound-guided arterial access can further improve safety by providing real-time target vessel visualization during needle entry, mitigating the risk of complications such as retrograde dissections, pseudoaneurysms, or insidious retroperitoneal bleeds at puncture sites. Distinguishing arterial pulsatility from venous compressibility using ultrasonography can help during cannulation, particularly during critical moments such as initiating extracorporeal membrane oxygenation in unstable patients when efficient first-pass success is paramount.

After identifying an entry point, micropuncture sets allow precise vessel entry. Alternative arterial access routes are considered when severe vessel tortuosity/calcification or small (<5 mm) intraluminal diameter preclude insertion of 14- to 16-Fr transcatheter aortic valve intervention (TAVI) delivery sheaths.^9^ For venous interventions (eg, transseptal mitral repairs), the common femoral vein or internal jugular vein may be targeted.

### Sheaths and Introducers

Short introducer sheaths (eg, Terumo GLIDESHEATH, Cordis AVANTI) provide portals for diagnostic catheters and guidewires (Table 1). Expandable sheaths (Edwards eSheath, Boston Scientific iSLEEVE, Cook Medical's Flexor) are used to accommodate TAVI or large stent grafts while minimizing trauma and blood loss. The GORE DrySeal or Medtronic Sentrant sheath are used to deploy the self-expanding Medtronic Evolut TAVI platform. The GORE DrySeal is also used during endovascular aortic repair (EVAR) and TEVAR, offering a hydrophilic coating that makes it useful for navigating complex aortic anatomy and the “DrySeal Valve,” which maintains hemostasis during device exchanges.

**Table 1:** Common sheaths and introducers used in transcatheter and endovascular interventions

Type of sheath	Name of device	Representative image
Introducer sheaths	Terumo GLIDESHEATH	https://www.terumois.com/products/access/glidesheath.html
	Cordis AVANTI	https://cordis.com/na/products/access/cardiology/avanti-introducer
Expandable sheaths	Edwards eSheath	https://www.edwards.com/healthcare-professionals/products-services/transcatheter-heart/transcatheter-sapien-3-valve-pulmonic
	Boston Scientific iSLEEVE	https://www.bostonscientific.com/en-IN/medical-specialties/structural-heart/tavi-portfolio.html
	Cook Medical Flexor	https://www.cookmedical.com/products/dfdfc483-b37b-49f2-8a78-937bf16ae831/
Deployment sheaths	Medtronic Sentrant sheath	https://www.medtronic.com/en-us/healthcare-professionals/products/cardiovascular/introducer-sheaths/sentrant-introducer-sheath.html
	Gore DrySeal	https://www.goremedical.com/sites/default/files/resources/pdf/2025-01/231302884-EN-DRYSEAL-Instruction-Card-Digital-FNL.pdf

### Vascular Closure Devices

Percutaneous vascular closure devices are used to seal arterial puncture sites after device removal, reducing the risk of pseudoaneurysms and facilitating early patient ambulation.^10^ These are either suture-mediated (eg, Abbott Perclose ProGlide) or collagen plug-mediated (eg, Angio-Seal, MANTA device).

### Managing Peripheral Access Complications

When gaining peripheral vascular access, the surgeon must be vigilant for complications such as hematoma, retroperitoneal hemorrhage, pseudoaneurysm, fistulas, and acute limb ischemia. Additional risks include access-site infections, improper plug apposition resulting in vessel rupture, and chronic vessel stenosis.^11^, ^12^, ^13^

#### Optimal arterial puncture technique

Ideally, CFA puncture should be performed between the origin of the inferior epigastric artery and the bifurcation of the CFA because proximal puncture elevates the risk of retroperitoneal hemorrhage and distal increases the risk of pseudoaneurysm, hematoma, and arteriovenous fistulas.^11^^,^^12^ Ultrasound guidance is preferred to fluoroscopy and palpation.^11^^,^^13^ Furthermore, micropuncture kits help reduce bleeding compared with traditional larger needles.^11^

#### Impact of procedure duration on vascular complications

Although extremities can generally tolerate reduced perfusion for up to 30 minutes, the risk of irreversible ischemic injury increases sharply after that.^13^ Therefore, prolonged sheath dwell times increase the risk limb ischemia and necrosis.^11^ Operators should be diligent in minimizing procedural duration, closely monitoring limb perfusion, and considering adjuncts like antegrade reperfusion sheaths or bypass circuits.^13^

#### Large-bore access considerations

Notably, large-bore sheaths can significantly impair distal limb perfusion in patients with small or calcified iliofemoral vessels.^13^ In high-risk patients, vigilant assessment, such as angiography via sheath sidearm or secondary access^11^^,^^13^ or monitoring via Doppler ultrasound during large-bore placement is strongly recommended.^14^

If ischemia does develop, management includes the following:

• Peel-away sheath technique: removing the outer sheath to reduce the profile (eg, with Impella devices);
• contralateral femoral-femoral bypass: temporary external bypass using connectors;
• ipsilateral antegrade bypass: direct access into the superficial femoral artery; and
• internal crossover bypass: perfusing through the profunda femoris if the superficial femoral artery is occluded.

#### Postprocedure access management

To minimize vascular complications and ensure hemostasis, a Perclose ProGlide (Abbott) closure device is often placed before upsizing sheaths beyond 8 F, a standard practice known as “preclosure.”^11^ This approach allows for safe sheath upsizing and effective hemostasis upon removal. In cases in which preclosure isn't possible, management options include balloon-assisted dry closure through contralateral access, plug-assisted closure (eg, the MANTA system), or hybrid techniques combining sutures and plugs for complex scenarios. Should a closure device fail, endovascular bailouts like stent placement or balloon tamponade are considered.^11^

### Principles of Contrast Injection and C-Arm Positioning in Angiography

Effective contrast enhancement is the cornerstone of endovascular imaging. To optimally visualize the proximal coronary arteries or the aorta, a target attenuation of 350 to 400 Hounsfield units is typically required.^14^ Careful mastery of several parameters is crucial to optimize imaging quality and minimizing contrast volume and associated complications:

• Contrast volume: A typical range is 50 to 100 mL, using contrast media with high iodine concentrations. Volume should be weight-adjusted, aiming for 245 to 370 mgI/kg to balance enhancement with safety.^14^
• Injection duration: An optimal injection time of 10 to 20 seconds allows thorough opacification while avoiding artifacts.^14^
• Flow rate: A robust arterial enhancement usually requires a flow rate exceeding 4 mL/s.^14^^,^^15^
• Saline flush: After contrast injection, a 30-mL saline flush helps centralize the contrast bolus, minimizes artifacts, and sharpens vessel delineation.^14^
• Scan timing: Two primary techniques synchronize scan acquisition with optimal vascular opacification:
  ○ Bolus tracking: Real-time monitoring of a region with scan initiation triggered when attenuation reaches 150 to 250 Hounsfield units.^14^
  ○ Test bolus: A smaller contrast injection (10-20 mL) is used to plot time-density curves and customize scan delay.^16^

#### Principles of C-arm positioning for optimal angiographic imaging

Precise C-arm positioning is vital for device deployment and minimizing radiation exposure and can be guided with preprocedural CT scans.^7^ To summarize, operators should:

• identify the aortic valve cusps and align them using a double-oblique CT plane;
• place fiducial markers at the nadir of each cusp;
• adjust the CT plane until the 3 cusps align in a straight line on the coronal view; and
• derive C-arm angulations: sagittal view determines cranial tilt (negative angle) or caudal tilt (positive angle), while coronal oblique view determines rightward (positive angle) or leftward (negative angle) rotation.^15^

Predefining these projections allows for coplanar alignment of the valve cusps, ensuring precise device deployment, reduced contrast injections, and shorter fluoroscopy times.^15^

## Basic Endovascular Terminology

### Guidewires

Guidewires range in size from 0.014 to 0.035 inches and vary in clinical applicability on the basis of wire core, coating, and tip design (Table 2).^17^^,^^18^ Their core material can be nitinol-based, which is more flexible or steel-based, which provides more structural support during device deployment but is prone to kinking and damage. Wire cores taper as you travel distally, increasing flexibility at the distal tip to reduce the risk of vessel trauma. Wires may have a hydrophobic or hydrophilic coating, the latter of which allows increased lubricity for advancement at the expense of decreased tactile sensation for the operator.^18^ Some common types of wires are as follows:

○ Diagnostic (working) wires: Used for initial percutaneous entry and travel up to the site of intervention, often featuring a soft, flexible tip to reduce risk of vascular damage and easily tracks across difficult angles. Examples include the Terumo GLIDEWIRE ADVANTAGE, Starter J wire, and The Wholey wire.
○ Support (stiff) wires: Support wires, such as the Lunderquist Extra-Stiff Wire, Safari Wire, Confida wire, or Amplatz Super Stiff wire, have a more rigid structure for crossing tortuous anatomy. They are also used during catheter exchanges when passing large sheaths and devices.
○ Specialty wires: Specialty wires, such as the Astato XS, feature strong, tapered tips that can more easily cross highly stenotic vessels, such as chronic total occlusions, but have increased risk for vessel dissection and perforation.
○ Hydrophilic wires: Hydrophilic coating lubricates the surface of a guidewire, reducing friction during vessel crossing and maneuvering. Examples include the Terumo GLIDEWIRE, which is highly advantageous in challenging tortuous anatomy.

**Table 2:** Reference sheet of wire, catheter, and balloon considerations for the cardiac surgeon

Category	Type	Examples	Uses/key features
Guidewires	Diagnostic/working wires	J-tipped wire, Terumo Glidewire Advantage, Wholey wire	- Initial percutaneous access and crossing - Soft, flexible tip to prevent vessel trauma - Available in various lengths and stiffness levels based on intended vessel and use
	Support wires	Lunderquist Extra-Stiff Wire, Amplatz Super Stiff	- High stiffness for crossing tortuous anatomy - Allows large-caliber catheter exchanges and deployment of devices and stents
	Specialty wires	Astato XT, ConfianzaPro	- Used for crossing high-grade plaque lesions and chronic total occlusions - Strong, tapered tips with high penetrance for improved crossability, torque control, and entry of microchannels - Increased risk of arterial perforation or creation of subintimal channels/dissections.
	Hydrophilic wires	Terumo GLIDEWIRE, Avigo	- Crossing tight stenoses, tortuous anatomy, and accessing steep branches during coronary and peripheral interventions - Hydrophilic coating increases lubricity allowing smoother crossing but lower operator tactile sensation
Catheters	Diagnostic catheters	Pigtail, Judkins Left/Right, Amplatz (AL/AR), multipurpose (MP), Cobra, Simmons	- Used during coronary angiography, aortography, ventriculography providing high-quality imaging or pressure measurements - Some feature preformed curves for selective vessel engagement during visceral (Cobra) or tortuous aortic arch (Simmons) interventions
	Guiding catheters	EBU, XB, Cook Ansel, Gore DrySeal	- Delivery of interventional devices (stents, balloons, endografts) - Feature larger internal diameters and stiffer structure to support device crossing - Improved contrast delivery and superior pressure monitoring capabilities during coronary (EBU, XB) or peripheral (Ansel, DrySeal) interventions
	Flush catheters	Cook Meducak Royal Flush Plus High-Flow, Cordis NYLEX	- Visualization of large vascular structures (aorta, superior/inferior vena cava) - Accommodates rapid, large-volume infusion of contrast material or therapeutic agents
Balloons	Pre/postdilation balloons	EMERGE (predilation), NC Quantum Apex (postdilation)	- Expanded either before or after stent/graft deployment to prepare lesion and optimize device expansion/apposition, respectively - Noncompliant design with high pressure-controlled inflation for precise sizing/adjustments
	Scoring/cutting balloons	AngioSculpt, Flextome	- Help combat atherosclerotic lesions that develop in native vessels (chronic total occlusion) or at the site of previous interventions (in-stent restenosis) - Compliant or semicompliant design with built-in cutting or scoring features to remove plaque as it travels
	Drug-coated balloons	IN.PACT Admiral, SeQuent Please	- Deliver antiproliferative therapeutic agents to reduce restenosis in peripheral or coronary arteries unsuitable for stenting or for treatment of in-stent restenosis
	Occlusion balloons	Fogarty, ER-REBOA	- Temporary vessel occlusion to achieve hemostasis - Compliant or semicompliant design to allow for vessel sealing with causing further trauma

*EBU*, Extra backup.

### Diagnostic and Guide Catheters

Catheters are designed to navigate specific vessels and allow contrast injections or device delivery (Table 2). Some common types of catheters are as follows:

• Pigtail catheters: Pigtails range in size from 5 to 8 Fr wide and 65 to 100 cm long, containing side holes and a curved tip to prevent vessel trauma during manipulation and high-pressure injections. They are commonly used for left ventriculography, aortic root angiography, or to measure pressures.
• Other diagnostic catheters: Judkins Left and Right catheters are 4- to 7-Fr diagnostic catheters primarily used in coronary and peripheral angiography, allowing selectively engagement, cannulation, and visualization of arterial structures. A multipurpose catheter ranges from 4 to 7 Fr and features a gentle 120° curve available in both single end-hole (MPA1) and dual-hole (MPA2) configurations, allowing various vessel engagement. Amplatz (AL/AR) catheters provide different curves for challenging anatomies. Cobra catheters have distinctive double-curve designs with angled tips for engaging visceral and bronchial vessels. For tortuous vascular anatomy such as in the aortic arch, operators may consider the Simmons catheters, which feature a reverse-curve design with preformed looping tip.
• Flush catheters: Flush catheters have thick walls to accommodate large infusion pressures and several small side holes to allow atraumatic rapid infusion of contrast into large vessels, such as the aorta or vena cava.^19^ Examples include the Cook Medical Royal Flush Plus High-Flow Catheter and Cordis NYLEX Angiographic Catheter.
• Guide catheters: Guide catheters are specialized catheters used in interventional procedures. Ranging from 6 to 8 Fr, they feature nontapered tips with larger internal diameters for enhanced device delivery and support. In addition, they have stiffer shafts, superior pressure-monitoring capabilities, and improved contrast delivery options. Examples include extra backup and XB catheters for coronary procedures and the Cook Medical Ansel for peripheral interventions.

### Balloons and Stents

When using balloon catheters, operators should note the rated burst pressure is the pressure up to which balloons can be inflated without bursting, and nominal pressure is the pressure the balloon inflates to when it reaches its standard diameter.^20^ Several functions are served by balloons (Table 1):

• Compliant balloons: Soft and pliable in nature, they can help score/remove thrombi and clots in vessels (scoring/cutting balloons) or temporarily occlude bleeding during arterial interventions (occlusion balloons). Examples include MicroVention BC0420C Scepter C and Z-MED Balloon Catheters (B. Braun Interventional).
• Noncompliant balloons: Stiffer when inflated, these can be used for predilation, postdilation, or as a scaffold during coronary stent or aortic endograft deployment. Rigid balloons can be further subclassified as singe-lumen monorail catheters with proximal wire extension or over-the-wire catheters, which has separate lumens for inflation and the wire itself.^20^ Examples of compliant balloon brands include MicroVention BC0420C Scepter C and Z-MED Balloon Catheters (B. Braun Interventional).
• Drug-coated balloons: These are specialized balloons coated in antiproliferative therapeutic agents and are often used to reduce atherosclerosis and vessel restenosis. Examples include the AGENT and SeQuent Please NEO drug-coated balloons, which can be used to treat restenosis after primary stenting.

Balloon expansion may be used to deploy TAVI valves (Edwards SAPIEN 3) or coronary stents (eg, Abbott XIENCE, Boston Scientific Promus). In TAVI, true Balloon and Z-MED balloons are widely used for pre- and postdilatation.

Similarly, balloon catheters are widely applicable in EVAR and TEVAR to deploy intraluminal endografts and improve graft apposition. When sizing, operators often elect to “oversize” the balloon to 110% to 120% of the native vessel diameter to ensure adequate sealing of the device and prevent the development of an endoleak.^15^ More recently, precannulating endografts followed by compliant balloon inflation at the sealing rings has been used to achieve optimal endograft size and seal on patients with complex aortic neck anatomy undergoing EVAR.^17^

### Contrast Agents

Contrast agents like iohexol (Omnipaque; GE HealthCare) and iodixanol (Visipaque; GE HealthCare) provide vessel and chamber opacification. Although both are nonionic and iodine-based, Omnipaque is a low-osmolar contrast medium, whereas Visipaque is an iso-osmolar contrast medium, having the same osmolality as blood. Visipaque is preferred in patients with renal insufficiency, because it may reduce nephrotoxicity and patient discomfort, although minimizing contrast load is critical.

## Wire and Catheter Manipulation Techniques

### Basic Wire Navigation

Under fluoroscopy, the operator advances the wire gently, rotating (“torquing”) it to steer it into desired branches. Hydrophilic wires glide smoothly but can perforate fragile arteries if not carefully handled. Stiff wires straighten vessel curves, facilitating device delivery but risk trauma if forced.

### Torque, Push, and Pull

A fine balance of torque, push, and pull manipulations allows for controlled navigation. Training with simulation and under experienced mentorship helps develop “wire feel.”

### Shaping the Wire and Choosing Catheter Curves

Manually shaping the wire tip, such as forming a slight J-curve helps navigate bifurcations and avoid injury. Matching catheter curves to patient anatomy (eg, JL4 for a typical left coronary system, multipurpose, or AR catheters for anomalous takeoffs) ensures stable engagement of target vessels.

## Endovascular Applications in Cardiac Surgery

### Transcatheter Valvular Interventions

#### Aortic valve

Balloon-expanding-valves (BEVs) (Edwards SAPIEN 3 and Myval [Meril Life Sciences]) are deployed in the intra-annular position and are often preferred for use in patients with conduction abnormalities as the result of the lower risk for need for pacemaker implantation.^21^ Conversely, self-expanding valves (Medtronic Evolut, ACURATE neo2 [Boston Scientific], Navitor [Abbott], ALLEGRA [Biosensors International]) can be deployed in the supra- or intra-annular position and are preferred in patients with smaller annuli because of their superior hemodynamics.^22^

Although the nuances of TAVI deployment are beyond the scope of this article, early trainees should be familiar with major steps, including the following^23^:

1. Percutaneous or surgical transfemoral access (alternate access includes transaxillary, trans-carotid, and transapical).^9^
2. Wire advancement of diagnostic pigtail catheter into noncoronary cusp and coplanar view establishment.
3. Aortic valve crossing, stiff wire exchange, and valve advancement under fluoroscopic guidance.
4. Valve deployment (rapid ventricular pacing is used in BEVs to reduce risk of valve migration).
5. Pre/postdilation as needed for heavily calcified and bicuspid valves.
6. Assessment of postdeployment hemodynamics.

During TAVI, 2 fluoroscopy views are used to optimize valve positioning^24^:

7. Three-cusp view: used primarily for BEVs and consists of aligning all aortic cusps in one fluoroscopic plane. This view is best for assessing coronary height before final deployment but carries an elevated risk of deeper valve implantation (Figure 1).

**Figure 1:**
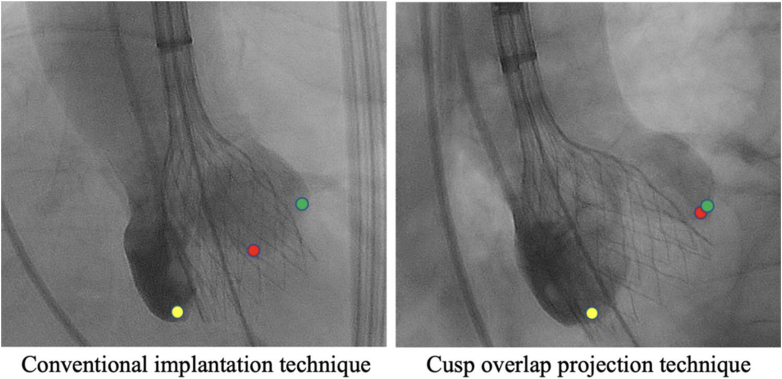
Three-cusp coplanar view versus 2-cusp overlap view with pigtail placed in the noncoronary cusp (*yellow*). The right and left coronary cusps are *red* and *green*.^24^
8. Two-cusp overlap view: used primarily for self-expanding valves, this view overlaps the left ∖and right cusps, shifting to a right anterior oblique-caudal view and isolating the noncoronary cusp. This helps eliminate parallax and facilitates greater implantation depth minimizing contact with the native conduction system.

Coronary obstruction is a rare but morbid complication of TAVI. Preprocedural CT planning can help evaluate the aortic valve, coronary ostia height, sinus of Valsalva diameters, and virtual transcatheter valve-to-coronary distance, with a distance <3 mm considered high risk for obstruction. Similarly, calculating the ratio of the distance from cusp to coronary ostium indexed to coronary artery diameter may also be used. During the procedure, interventionalists should ensure proper alignment of prosthetic valve commissures with native aortic valve commissures. Intravascular ultrasound may also be used to assess coronary ostia. High-risk patients may receive protective coronary stenting or adjunctive leaflet reduction with the BASILICA (Bioprosthetic or Native Aortic Scallop Intentional Laceration to prevent Iatrogenic Coronary Artery obstruction) procedure.^25^ If coronary obstruction does occur, percutaneous angioplasty or stenting may be used.

Although most TAVIs can be complete with intraprocedural fluoroscopy and transthoracic echocardiography under conscious sedation, transesophageal echocardiography (TEE) remains in patients with severe renal insufficiency in whom contrast use needs to be minimized or in complex procedures requiring enhanced imaging precision, such as valve-in-valve cases.^18^

#### Mitral valve

Transseptal puncture is a critical step in gaining access to the left atrium during mitral valve interventions. Preprocedural CT imaging may assist with procedural simulation, including planning the ideal height, angle of entry, and C-arm orientation.^26^ Right femoral venous access is followed by advancement of a sheath and dilator into the superior vena cava. A Brockenbrough needle is then introduced and withdrawn under fluoroscopic and TEE guidance to enter the fossa ovalis. Intraprocedural imaging guides needle trajectory and confirm tenting before puncture. Steerable sheaths and radiofrequency-assisted needles may be used in challenging anatomies.^26^

#### Transcatheter edge-to-edge repair

Transcatheter edge-to-edge repair is an established treatment option for patients with severe mitral regurgitation unamenable to surgery. Modeled after the surgical Alfieri stitch technique, the MitraClip system (Abbott) mechanically approximates the anterior and posterior mitral leaflets, creating a double-orifice valve and reducing mitral regurgitation.^27^ The clip delivery system allows precise leaflet grasping through independently operated grippers.

Alternatively, the PASCAL transcatheter valve repair system (Edwards Lifesciences) is an emerging option for transcatheter edge-to-edge repair with enhanced leaflet grasping, stress distribution, and navigation control.^28^ The device consists of a central spacer that fills the regurgitant orifice, broad nitinol paddles for distributing force across the leaflets, and independently controllable clasps for staged or simultaneous leaflet capture. These features help facilitate leaflet approximation, particularly in complex anatomies such as large coaptation gaps, Barlow's disease, and flail segments.^28^

#### Tricuspid valve

For tricuspid disease, the Edwards EVOQUE has shown high rates of procedural success and symptom improvement, alongside low rates mortality and complications in 1- and 2-year follow-up. The PASCAL system has also been successfully used with its flexible design and independent leaflet capture advantageous in the large and fragile tricuspid valve.^21^ In addition, devices such as the TriClip (Abbott) are in trial for transcatheter tricuspid repair.

### Thoracic Endovascular Aortic Repair

Stent graft systems like Gore TAG Conformable Thoracic Stent Graft or Medtronic Valiant Captivia are commonly used for thoracic aneurysms, dissections, or penetrating ulcers. In addition, new thoracoabdominal branch and zone 2 thoracic branch endoprostheses are emerging options for patients. The surgeon must understand device sizing and appreciate landing zones.^22^ Preprocedural CT planning determines feasibility and the need for adjunct maneuvers, such as chimney or fenestrated grafts for branch preservation.

Like TAVI, trainees should refer to previously published literature highlighting the nuances of TEVAR and learn major procedural steps, including the following^29^:

1. Percutaneous or surgical access of femoral artery.
2. Wire advancement of pigtail catheter into aorta for baseline angiography.
3. Stent graft deployment over stiff wire under fluoroscopic guidance (blood pressure should be lowered to <80 mm Hg using vasodilators or rapid right ventricular pacing to reduce risk of downstream displacement).
4. Postdeployment ballooning as needed for adequate device sealing.
5. Completion angiography to exclude endoleaks and visceral obstruction.

During TEVAR, a sufficient proximal landing zone of >2.5 cm of healthy native aorta is necessary to exclude pathology and reduce the risk of endoleak.^30^ Some centers have published novel approaches for managing complex distal aortopathies, including the use of physician-modified endografts and branched stented anastomosis frozen elephant trunk repair (B-SAFER)^23^ and hybrid open and endovascular aortic surgery.^31^

## Adjunctive Imaging Technology

### Echocardiographic Guidance

During structural interventions, TEE or intracardiac echocardiography offers real-time anatomical guidance. TEE plays a crucial role in guiding valve interventions, confirming device positioning, leaflet coaptation, and/or hemodynamics. 3D TEE provides spatial orientation of valve apparatus and paravalvular leaks. Intracardiac echocardiography catheters, such as the ACUSON AcuNav, are particularly useful in transseptal interventions, providing close-range imaging without the need for general anesthesia.

### CT and 3D Reconstruction

Because endovascular approaches limit intraoperative visualization compared with open surgery, high-quality imaging and anatomical reconstruction is paramount for operative planning. CT planning before TAVI and TEVAR helps assess anatomical suitability. Professional software like 3mensio or HeartNavigator help generate 3D reconstructions of patient anatomy to simulate device placement and predict potential complications, such as coronary sinus obstruction (TAVI) or visceral occlusion (TEVAR/EVAR).

## Training Pathways and Skill Acquisition

### Curriculum Integration

As the field of cardiac surgery evolves, training programs must integrate transcatheter and endovascular training into their curricula. Accordingly several, particularly integrated 6-year programs now include rotations in the catheterization laboratory, structural heart, and cardiovascular imaging as well as structured didactics on device and imaging basics.^25^^,^^26^ Endovascular simulation platforms (eg, Mentice VIST, Simbionix ANGIO Mentor) can also help residents develop hand-eye coordination, wire-handling skills, and device familiarity.

### Mentorship and Proctorship

Working under the guidance of experienced operators accelerates skill acquisition. Observing device deployment, troubleshooting techniques, and technical nuances is invaluable. Attending dedicated workshops, visiting high-volume centers, and seeking formal proctorship enhances proficiency.

## Conclusions

The integration of endovascular interventions is reshaping the specialty and expanding the armamentarium of the cardiac surgeon. For trainees and early-career surgeons, gaining experience with these tools will prepare them to take on central roles in multidisciplinary teams and serve as catalysts in merging the best of surgical precision with minimally invasive innovation.

## Conflict of Interest Statement

M.A.: consultant for Gore Medical and Medtronic Endovascular. C.K.M.: Consultant for Gore Medical and Terumo. B.T.: Consultant for Gore Medical. All other authors reported no conflicts of interest.

The *Journal* policy requires editors and reviewers to disclose conflicts of interest and to decline handling or reviewing manuscripts for which they may have a conflict of interest. The editors and reviewers of this article have no conflicts of interest.
